# Understanding Gender-Specific Daily Care Preferences: Topic Modeling Study

**DOI:** 10.2196/64160

**Published:** 2025-05-29

**Authors:** Kyungmi Woo, Se Hee Min, Aeri Kim, Subin Choi, Gregory L Alexander, Terrence O’Malley, Maria D Moen, Maxim Topaz

**Affiliations:** 1 College of Nursing Seoul National University Seoul Republic of Korea; 2 Visiting Scholar Columbia University School of Nursing New York, NY United States; 3 School of Nursing University of Pennsylvania Philadelphia, PA United States; 4 The Research Institute of Nursing Science Seoul National University Seoul Republic of Korea; 5 School of Nursing Columbia University New York, NY United States; 6 Corresponding Faculty Harvard University Boston, MA United States; 7 Innovation and External Affairs MyDirectives Inc Nashville, TN United States; 8 Columbia University Data Science Institute New York, NY United States

**Keywords:** care preference, patient-centered care, quality of life, data mining, topic modeling, gender differences, online platform, KNIME

## Abstract

**Background:**

Daily preferences are a reflection of how adults wish to have their needs and values addressed, contributing to joy and satisfaction in their daily lives. Clinical settings often regard older adults as a uniform group, neglecting the diversity within this population, which results in a shortfall of person-centered care that overlooks their distinct daily care preferences. At the heart of person-centered care lies the imperative to comprehend and integrate these preferences into the care process. Recognizing and addressing gender differences in older adults is critical to customizing care plans, thereby optimizing quality of life and well-being for individuals. This study addresses the need to understand the diverse daily care preferences of adults, particularly among older populations, who represent a growing demographic with unique needs and interests.

**Objective:**

This study aims to identify and analyze the key themes and daily care preferences from unstructured adult text narratives with a focus on uncovering gender-specific variations.

**Methods:**

This study used 4350 deidentified, unstructured textual data from MyDirectives (MyDirectives, Inc), an interactive online platform. Advanced topic modeling techniques were used to extract meaningful themes, and gender-specific term frequency and distribution were examined to identify gender differences in these elements.

**Results:**

The study sample included 2883 women (mean age 63.02, SD 13.69 years) and 1467 men (mean age 67.07, SD 11.73 years). Our analysis identified six major themes: (1) “entertainment” (12.14%, 528/4350), (2) “music” (10.39%, 452/4350), (3) “personal interests and memories” (38.18%, 1661/4350), (4) “intimate relationships” (14.92%, 649/4350), (5) “natural comforts” (16.18%, 704/4350), and (6) “emotional, cultural, and spiritual foundations” (8.18%, 356/4350). Gender differences were evident: women were more likely to express preferences for “personal interests and memories” (40.7% vs 33.3%), “natural comforts” (18.4% vs 11.9%), and “emotional and spiritual foundations” (9.3% vs 6.1%) than men. Men expressed stronger preferences for “entertainment” (18.1% vs 9.1%) and “music” (16.8% vs 7.2%). Common terms across all participants included “dog,” “love,” “friends,” and “book.” Notably, the study revealed significant gender differences in daily care preferences, especially regarding familial relationships and entertainment choices.

**Conclusions:**

The findings underscore the importance of recognizing individual daily care preferences in person-centered care, particularly regarding gender. Understanding these preferences is crucial for improving care quality and patient satisfaction, thereby enhancing the overall quality of life for adults receiving care across our health care system.

## Introduction

Older adults live relatively healthier lives with longer life expectancies thanks to recent advances in medical science and technology [1]. However, older adults continue to face significant health care challenges, including a persistent disability burden [2] and reliance on various care services, such as hospitalization and long-term care assistance with daily living activities [3]. Despite these complexities, older adults are often treated as a homogeneous group in clinical settings [4] rather than a heterogeneous group of individuals, leading to a lack of person-centered care that fails to consider their unique daily care preferences [5-7]. This oversight results in suboptimal health outcomes, higher rates of overtreatment and unwanted care [8], and increased patient dissatisfaction [5-7]. Furthermore, the rising number of younger adults in various care settings [9] underscores the importance of understanding daily care preferences across all age groups for delivering high-quality, person-centered care [10].

Daily preferences reflect how adults prefer their needs and values met for those aspects of living that bring them joy and happiness in their daily lives [11,12]. When daily preferences are fulfilled, adults experience a sense of subjective well-being and happiness, creating motivation to actively engage in life [11,13]. Furthermore, happiness has been associated with a reduced likelihood of all-cause mortality and better health outcomes such as functional independence, especially among older adults [14]. Thus, the tenet of person-centered care is understanding adults’ daily preferences and effectively incorporating them into care [15,16]. Such delivery of person-centered care will enable better health-related quality of life for adults regardless of their care setting [15,16].

Regardless of age, daily care preferences are highly likely to be individualized and diverse based on individual values, sociodemographic factors, and care settings [11,12]. A recent study found significant gender differences in everyday living preferences among older adults [17]. For example, older women adults had a stronger daily preference in four areas, including having a caregiver of the same gender and a desire to engage in volunteer work, household tasks, and decorating. In contrast, older adults’ men preferred a hands-on approach when learning new things and did not emphasize volunteer work [17]. Another study found a significant difference in future living arrangements for middle-aged adults based on gender [18]. For example, middle-aged women were more likely to prefer to live in a nursing home when they became older, with a lack of preference to live in the community compared with middle-aged men [18]. Recognizing these individual daily care preferences is crucial, and a key aspect to consider is the significant impact of gender on adults’ daily care preferences. Understanding these gender differences among older adults is essential to effectively tailor care plans and ensure the highest quality of life and well-being for adults of all ages [12].

This retrospective study used deidentified data from MyDirectives, Inc (formerly ADVault, Inc) [19], an interactive and innovative online platform that easily stores, responds to queries, and retrieves health care information between health care providers and patients in an interoperable, standards-based manner. The purpose of MyDirectives, coupled with the mission of the Moving Forward Coalition [20], is to enable the delivery of person-centered care through understanding adults’ daily care preferences and incorporating them into person-centered care plans. The MyDirectives application asks adults to directly describe their daily care preferences in free text, thus providing more abundant and reliable information than simple, categorized responses from the survey data. Using MyDirectives data coupled with topic modeling, a type of natural language processing (NLP) technique, can uncover latent topics within a large corpus of texts that contain information on adults’ daily care preferences [18]. Understanding meaningful topics of daily care preferences can help health care professionals better incorporate them into a plan of care, improving the provision of person-centered care, the quality of patient care, and health outcomes.

This study identifies and analyzes key themes and daily care preferences from unstructured adult text narratives. Additionally, this analysis explores the variances and commonalities in these themes between genders, offering a comprehensive understanding of daily care preferences as reflected in the narratives.

## Methods

### Data Source and Sample

The current study used deidentified, unstructured textual data extracted from MyDirectives, a sizable, interactive online platform facilitating seamless access to health-related information and fostering communication between health care providers and patients. A random sample encompassing 10,000 unique records was derived from community-dwelling adults across the United States. This investigation examined responses to the following targeted query:

My Likes/Joys: Describe the things that bring you joy. Photographs or other items you would like to have nearby, or music you’d like to hear. A favorite pillow, a night light, or your favorite flowers.

Furthermore, participant gender and age (in 10-year increments) were acquired by administering standardized questionnaires embedded within the MyDirectives interface.

### Data Preparation

**Figure 1:**
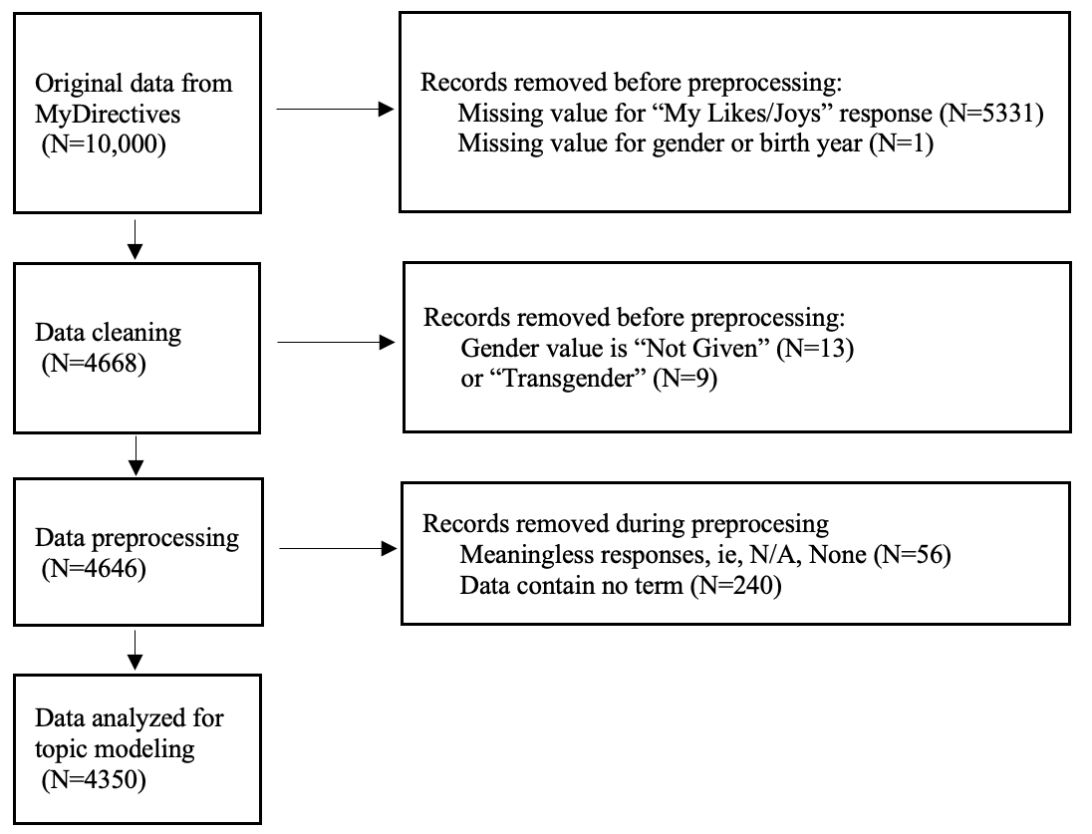
Flowchart illustrating the data selection process for the topic modeling study on daily care preferences among US adults. The figure outlines participant inclusion criteria, exclusion steps, and the final sample (N=4350) used for natural language processing analysis.

From 10,000 records, 4646 records were processed after excluding 5331 records with missing responses in the daily care preferences, 1 record with missing gender or birth year information, and 22 records with gender answered as “transgender” (N=9) or “not specified” (N=13). The exclusion of records from the transgender group (N=9) was due to the small sample size, which would not provide sufficient data for robust analysis using topic modeling techniques and gender differences. In addition, 296 records with irrelevant responses (eg, “N/A” and “None”) were removed. As a result, a final dataset of 4350 records was used for the latent Dirichlet allocation (LDA) topic modeling (Figure 1).

### Preprocessing

In this study, we applied standard NLP procedures to prepare textual data for analysis. The process involved several steps:

1. Removal of punctuation and numerical characters: We stripped away all punctuation marks and numbers from the text, as these elements are not required for our NLP analysis.
2. Conversion to lowercase: The entire text was converted to lowercase to ensure uniformity, so the system treats words such as “Health” and “health” the same.
3. Tokenization: This step involved breaking down the text into individual elements, or “tokens,” typically words or phrases.
4. Lemmatization: We transformed words into their base or root form. For instance, variations of a word such as “running” were reduced to their simplest form, “run.”
5. Elimination of common English stop words: Commonly used English words such as “I,” “my,” “on,” “of,” “what,” “and,” “the,” and “from” were removed. These words, often called stop words, are usually excluded in NLP as they offer little value in understanding the key topics of the text.

### Topic Modeling

The study used LDA topic modeling using the Konstanz Information Miner (KNIME) Analytics Platform (version 4.7.2; KNIME) [21]. Python (version 3.10.12; Python Software Foundation) was used to determine the most appropriate number of topics. LDA topic modeling, an unsupervised machine learning technique and a generative probabilistic algorithm, aims to uncover the number of topics (K) within extensive document collections [22]. A random mixture of latent topics represents these documents, defining the distribution of topics within a specific document (α) and the distribution of words for a given topic (β) [22,23]. The latent topic themes are not directly observable and thus necessitate inference based on their constituent words. Researchers specify K as input hyperparameters (Multimedia Appendix 1). This study determined the optimal K, as described in the “Choice of Optimal K for LDA Topic Modeling” section [24,25]. Previous studies have effectively used similar parameters in LDA topic modeling to reveal concealed themes across diverse research domains within large corpora, including health care–related patient feedback, newspapers, and open-ended survey responses [26-28]. An expert panel of 7 specialists in relevant fields comprehensively assessed the topic modeling results. The panel included 4 researchers (KW, AK, MT, and SC) with expertise in topic modeling, 3 (KW, SHM, and AK) with a background in psychological health research among older adults, and 1 (KW) with experience in care planning. In addition, we had experts (KW and AK) with a background in nursing home care, MT specializing in informatics, and GLA, TAO, and MDM, who are authorities in nursing homes and long-term care and are leading efforts in nursing home reform through the Moving Forward Coalition.

### Choice of Optimal K for LDA Topic Modeling

To ensure our study was accurate, we needed to determine the best number of topics to use in our analysis, a process known in technical terms as selecting the optimal “K” for LDA topic modeling. This step is crucial because too many topics can make the analysis confusing and overlapping, while too few can make the topics too broad and unclear. We used several methods to find the right balance:

1. Perplexity measure: This helped us understand how well our model could predict the text. We looked for the lowest perplexity, meaning the model was more accurate in understanding the text [29].
2. Coherence score: Using Python Gensim (RARE Technologies Ltd) [30], we measured how logically the words within each topic related. Higher scores meant the topics made more sense, such as the words “nurse,” “care,” “patient,” and “hospital” being grouped.
3. Elbow method: We also used the elbow method, which helped us visualize the best number of topics by looking at how texts grouped at different numbers of topics [31].

By combining these methods, we could find the most effective number of topics for our study, ensuring our analysis was accurate and understandable [32].

### Gender Differences Throughout the Keywords and Themes

**Figure 2:**
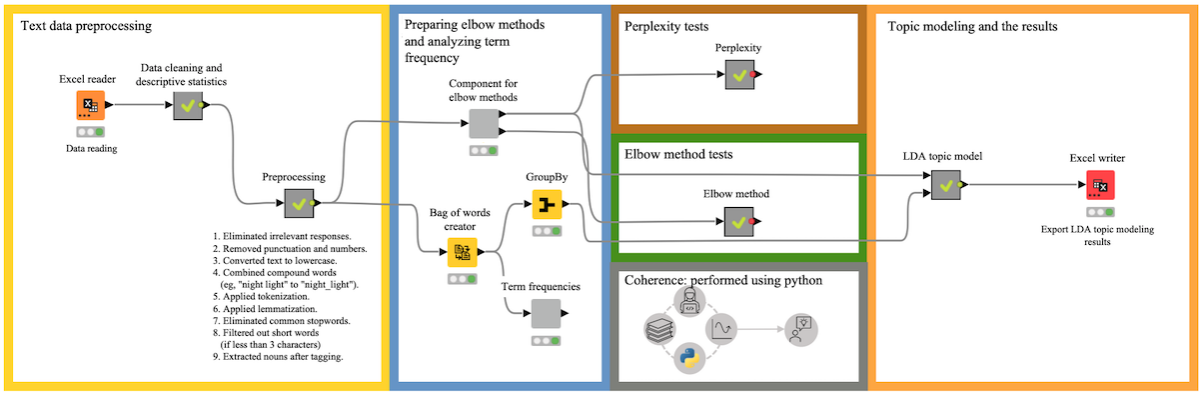
The Konstanz Information Miner workflow of topic modeling applied to unstructured text responses collected via MyDirectives. The process includes tokenization, lemmatization, and removal of stopwords before topic modeling using latent Dirichlet allocation. Tokenization refers to the process of breaking down a stream of text into smaller units called tokens, typically words. Lemmatization is the process of converting variations of a word that reflect tense or number to its root form, called a lemma. For instance, the words “writes,” “wrote,” and “written” would all be lemmatized to “write.” Stopwords are words that are deemed to have little to no intrinsic value for understanding the essence of the content. These commonly include words such as “I,” “my,” “on,” “of,” “what,” “and,” “the,” and “from.” Model optimization was guided by perplexity, coherence, and the elbow method. LDA: latent Dirichlet allocation.

A term frequency analysis was used to identify gender differences in the usage and prevalence of keywords associated with daily care preferences. We extracted and normalized the frequencies of the top 40 words for each gender, and these normalized values were visualized in heatmaps, allowing for a visual comparison of term usage priorities and intensities between genders. Notably, the prominence of the top 4 words (“music,” “family,” “photo,” and “picture”) in both men’s and women’s datasets was primarily due to their inclusion as examples within the *My Likes/Joys* open-ended question. Consequently, their consistently high occurrence dampened the variability in the heatmap. Therefore, after excluding these 4 terms, we investigated gender differences in daily care preferences by comparing the heatmaps generated from the remaining top 40 words. Figure 2 represents the procedural workflow executed in KNIME, outlining the steps of the actual research. In addition, a radar chart, also called a spider chart, is used to visualize the comparison of gender distributions across themes, providing a comprehensive view of gender patterns and differences on a single chart. A radar chart displays each value of a variable along axes arranged at equal angles around a central point. Within this structure, each variable is plotted along its corresponding axis, forming a polygon, enabling the comparison and visualization of multivariate data. The central point represents the smallest value and extends outward to represent larger ones.

### Ethical Considerations

This study was reviewed and deemed exempt from institutional review board review by Columbia University (exemption number AAAU5320), under ethical guidelines for secondary analysis of deidentified data. As the dataset consisted of deidentified data provided under a formal data use agreement, no additional informed consent was required for this analysis. The original data collection obtained appropriate consent from the users, and the secondary use was authorized by the data provider, MyDirectives, Inc. To protect participant privacy and confidentiality, all data were fully deidentified before analysis, and no personally identifiable information was available to the research team at any stage. No compensation was provided, as this study did not involve direct contact with participants. Finally, no images or figures in the paper or Multimedia Appendix 1 contain any information that could be used to identify individual participants.

## Results

### Participant Characteristics and Text Data

The LDA topic modeling analyzed 4350 free-text responses regarding daily care preferences among adults. The average age of the 4350 participants was 64.38 (SD 13.20) years, consisting of 2883 women (mean age 63.02, SD 13.69 years) and 1467 men (mean age 67.07, SD 11.73 years). Regarding the free-text data, the average word count was 18.92 (SD 24.11) with an average character count of 107.18 (SD 131.90).

### Optimal Number of Topics

**Figure 3:**
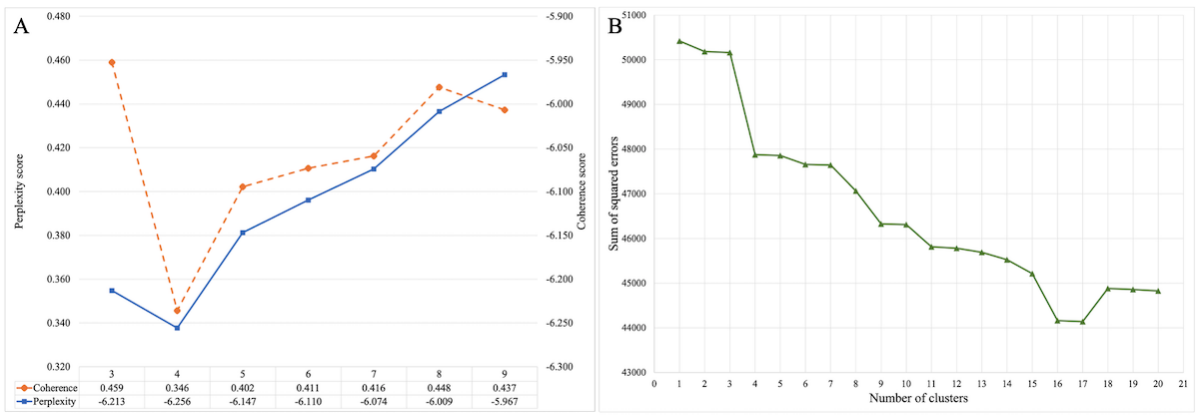
Determining the optimal number of topics for latent Dirichlet allocation modeling. (A) Coherence and perplexity scores across topic counts. (B) Elbow method illustrating model fit. Analyses were conducted using Python and Konstanz Information Miner.

The determination of the optimal number of topics involved several evaluation methods, including perplexity, coherence, and the elbow method. Figure 3 shows the results of these evaluation approaches. Although K=4 shows the lowest perplexity score, it also yields the lowest coherence score (Figure 3A), suggesting potential inconsistencies and reduced interpretability within the model. As K increased to K=8, the topic coverage declined while consistency improved. The elbow method illustrated a sharp shift in the sum of squared errors values as K transitioned from 3 to 4, remaining stable as K ranged between 5 and 7 (Figure 3B). Considering these findings, individual LDA topic models were constructed at K=5, 6, and 7, each comprised of 15 keywords. The research team conducted iterative reviews to evaluate interpretability and distinguishability, concluding that 6 topics were the most appropriate. For the LDA topic model with 6 topics, α (0.31) and β (0.91) values were selected as hyperparameters, exhibiting the highest coherence.

### Topic Modeling

Table 1 displays the identified themes, their top 15 keywords, and the respective proportions for each topic, extracted through LDA topic modeling of the free-text responses regarding daily care preferences in care among adults. The multidisciplinary team collaboratively identified six dominant themes: (1) “entertainment,” (2) “music,” (3) “personal interests and memories,” (4) “close relationships,” (5) “natural comforts,” and (6) “emotional and spiritual grounding.” Notably, the results showed that the theme of “Personal interests and memories” (Topic 3) accounted for the largest proportion of daily care preferences at 38.18%. “natural comforts” (topic 5) and “close relationships” (Topic 4) followed with 16.18% and 14.92%, respectively.

**Table 1:** Latent Dirichlet allocation topic modeling results based on 4350 free-text responses from US adults using MyDirectives, an online advance care planning platform.

Rank	Topic 1: Entertainment		Topic 2: Music			Topic 3: Personal interests and memories			Topic 4: Close relationships			Topic 5: Natural comforts			Topic 6: Emotional and spiritual grounding	
	Keyword	Weight (528/4350, 12.14%)	Keyword	Weight (452/4350, 10.39%)	Keyword		Weight (1661/4350, 38.18%)	Keyword		Weight (649/4350, 14.92%)	Keyword		Weight (704/4350, 16.18%)	Keyword		Weight (356/4350, 8.18%)
1	Music	500	Music	1063	Music		2915	Family		568	Music		712	Music		1740
2	Book	429	Country	456	Family		2502	Picture		508	Flower		418	Flower		367
3	Movie	379	Rock	221	Picture		1145	Photo		446	Dog		350	Love		182
4	Cat	180	Jazz	151	Photo		1020	Dog		420	Pillow		325	Dog		123
5	Phone	144	Song	102	Flower		818	Friend		359	Time		312	Time		122
6	Reading	109	Piano	91	Dog		374	Child		353	Blanket		247	Gospel		116
7	Video	105	Roll	81	Pillow		371	Time		340	Animal		236	Joy		109
8	Game	95	Blues	74	Friend		349	Grandchild		300	Window		228	Rose		100
9	Sport	92	Pop	67	Country		347	Joy		239	Bed		198	Song		98
10	Computer	87	Pink	65	Gospel		332	Husband		208	Nature		193	Life		92
11	Radio	86	Guitar	54	Pet		281	Son		195	Sound		152	People		80
12	Football	86	John	49	Photograph		273	Daughter		192	Light		148	Day		78
13	Comedy	81	Play	49	Rose		242	Wife		172	Bird		147	Color		75
14	Puzzle	75	Folk	46	Kid		232	Life		172	Ocean		138	God		54
15	Access	70	Beethoven	46	Night light		220	Sister		162	Air		131	Food		51

The first theme was identified as “entertainment.” The keywords, such as “music,” “book,” “movie,” “video,” “game,” “sport,” “football,” “comedy,” and “puzzle,” indicate the diverse range of entertainment preferences. This theme involves various activities, including listening to music, reading books, watching movies, and solving puzzles. Additionally, the inclusion of keywords such as “phone,” “computer,” and “radio” indicates the technological media used to access entertainment.

The analysis revealed the second theme, labeled “music.” Notably, “music” stands out as the most prominent keyword across all themes, except “close relationships” (Topic 4). The extracted keywords represent various music genres, including “country,” “rock,” “jazz,” “rock and roll,” “blues,” and “pop.” Specific musical instruments such as “piano” and “guitar” are also mentioned alongside favored composers and singers. Hence, this theme highlights a broad spectrum of musical interests, genres, instruments, and preferences for artists or composers.

The third theme, “personal interests and memories,” accounts for the largest proportion of all topics. It includes a wide range of personal interests, such as “music,” “flowers,” “dogs,” “pillows,” and “night lights.” Moreover, it emphasizes the importance of valuing memories, indicated by keywords related to “family,” “friends,” “pets,” and “kid,” revealing a wish to preserve these moments through “photographs.”

The fourth theme, “close relationships,” explores the importance of emotional ties with loved ones. It includes keywords such as “family,” “dog,” “friend,” “child,” “grandchild,” “husband,” “son,” “daughter,” “wife,” and “sister.” This theme encapsulates the essence of cherished relationships, underscoring a preference to share moments of “joy” and precious “time” with close individuals.

The fifth theme, “natural comforts,” focuses on seeking solace, tranquility, and comfort within nature. This theme is characterized by nature-related keywords, including “flower,” “dog,” “animal,” “light,” “sound,” “bird,” “ocean,” and “air,” which convey a sense of the natural world and emphasize a preference for the tranquil atmosphere they provide. Additionally, it indicates a preference for comfort when keywords such as “music,” “pillows,” “light,” and “blankets” are accompanied by “time,” “bed,” and “window.”

Finally, the sixth theme, “emotional and spiritual grounding,” is centered around keywords such as “music,” “love,” “dog,” “time,” “gospel,” “joy,” “song,” “people,” “day,” and “god.” This theme reveals the significance of emotional connections by emphasizing love and joy, portrayed through music, companionship with pets, and interactions with people. Furthermore, it extends into the spiritual realm, bringing out the essence of joy and love through the gospel and God.

### Gender Differences in Daily Care Preferences

**Figure 4:**
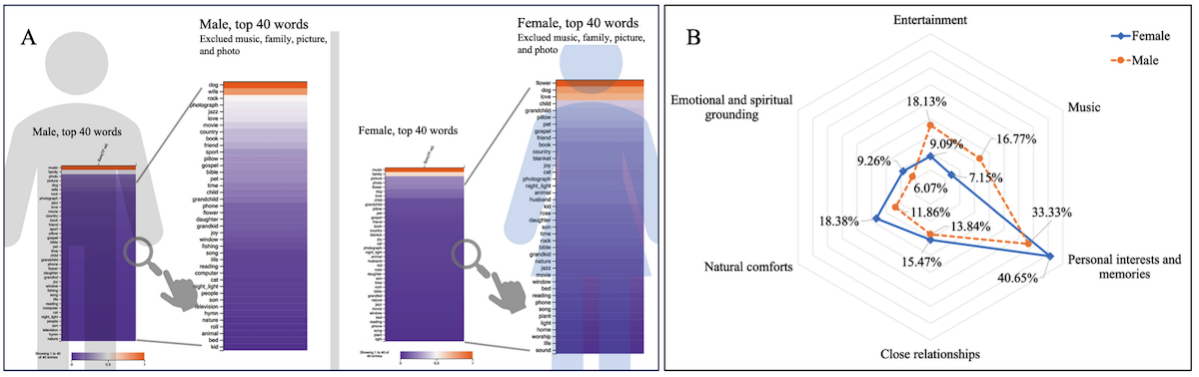
Gender-based keywords and thematic differences in daily care preferences among US adults. (A) Heat map of normalized term frequencies for the top 40 most common words by gender. (B) Radar chart comparing the distribution of 6 major themes across women and men subgroups, based on topic probability weights.

The gender differences in daily care preferences associated with a better quality of life were examined and displayed in Figure 4A, using the frequency of the top 40 words by each gender in the sample. The top four words, “music,” “family,” “photo,” and “picture,” were predominant in both genders. Except for the top four words, “dog,” “love,” “friends,” and “book,” they were the common, frequent top 10 words in men and women. Men more commonly mentioned hobby-related words such as “movie,” “sports,” “fishing,” “computer,” and “television,” while for women, “blanket,” “joy,” and “phone” were more prevalent. These findings were further supported by the results shown in the radar chart in Figure 4B. Women demonstrated higher daily care preferences for themes associated with “personal interests and memories,” “natural comforts,” and “emotional and spiritual grounding” than men. meanwhile, men preferred “entertainment” and “music” themes more than women.

## Discussion

### Principal Findings

To the best of our knowledge, this is the first study to identify keywords and themes that capture the daily care preferences of adults residing in the United States, specifically focusing on examining gender differences based on word frequency and themes. Using LDA topic modeling, we extracted six distinct themes: (1) “entertainment,” (2) “music,” (3) “personal interests and memories,” (4) “close relationships,” (5) “natural comforts,” and (6) “emotional and spiritual grounding.” in addition, we found gender differences in specific familial relationships (eg, “close relationships”) and their preferred entertainment activities.

The first theme, “entertainment,” emerged as a broad topic in our study, incorporating traditional activities such as reading, sports, and music. About gender differences within this theme, it was notable that the entertainment theme accounted for nearly twice the proportion of daily care preferences for men compared with women (18.13% vs 9.09%). This marked the second-largest gender difference among the themes. Moreover, solitary activities such as watching movies and reading books were more prominent in men than women. In contrast, women exhibited a stronger tendency toward interpersonal connections, expressing emotions (ie, love and joy) through words. The “Entertainment” theme, which encompasses technological terms such as “phone,” “video,” “game,” and “computer,” suggests a significant digital dimension to entertainment activities. Recent studies exploring technology in adult entertainment, including virtual reality, have suggested potential positive impacts on physical and psychological health [33,34]. The current study noted a clear preference for electronic devices such as computers, televisions, and phones among men compared with women. Meanwhile, in the context of nursing homes, research has shown that men residents were less satisfied with the activities provided to them than women, indicating a significant disparity in satisfaction between genders [35]. This disparity may suggest a lack of integration of gender-specific entertainment preferences into care and care plans. Consequently, it is important to consider gender differences when providing entertainment in adult care, as supported by studies demonstrating varied attitudes toward communication and entertainment devices between genders [36,37].

The music theme encompassed various genres, indicating a broad spectrum of musical preferences among adults. This thematic prominence of “music” within the adult preference spectrum has significant implications for person-centered care, especially in nursing homes. Recognizing the diversity of musical interests can guide the development of personalized care plans that use music therapeutically to help improve health status by reducing depression, agitation, behavior problems, blood pressure, anxiety, and pain [38-40]. The perceived importance of music in the relationship between music and pain management could serve as a mechanism for better pain management, care planning, and quality of life [40]. Gendered preferences within this music theme were particularly pronounced: men showed a stronger tendency toward “rock music,” while women showed a preference for “country songs.” These results revealed that “music” had a significantly higher share of interest among men than women in their preferences, and the distinct music styles preferred by men and women align with findings from existing research [40,41]. Based on this finding, care providers may need to assess and provide tailored musical activities to enhance health-related quality of life for nursing home residents.

The third theme, “personal interests and memories,” constitutes the majority of responses, underscoring its significance for both men and women as the most meaningful aspect of their daily care preferences. Accordingly, it becomes evident that integrating individual preferences and histories into daily care is essential, ranging from music and family to photos, botanical elements, beloved animals, and comforting items such as pillows and night lights. More specifically, in a person-centered care framework, Morgan and Yoder [42] mentioned that recognizing each resident’s unique histories and preferences and incorporating these individualized elements into care plans are crucial. Recognizing preferences to support person-centered care may help to improve a sense of dignity, autonomy, and quality of care while reducing behavioral disturbances [42-44]. When individuals encounter familiar objects and themes from their personal histories, such as a favorite family photo or a beloved flower, it can stimulate memories and emotions, enhancing cognitive engagement and emotional satisfaction [45]. Notably, our findings highlight a gendered pattern in these interests and memories, where men were prominently associated with keywords such as music, dogs, and photographs, while women were more connected to keywords suggesting a familiar atmosphere, such as flowers, pillows, blankets, and night lights. By considering these preferences, staff can cultivate a home-like care environment that not only meets physical health needs but also nourishes the psychological, emotional, and unique aspects of a person’s life [46].

The fourth topic, “close relationships,” highlights the essence of human connection through keywords such as family, friends, and pets. Interestingly, while “family” emerged as an important element across genders, there were notable differences in the priority of specific family members. Men frequently mentioned “wife,” whereas women placed “child” and “grandchild” above “husband” in prominence. This suggests gender-based differences in the perception and valuation of family roles and relationships [47,48]. In long-term care contexts, these insights are vital for person-centered care, as families often play an important role in care planning. Care plans that involve family members not only reduce depression but also enhance their sense of well-being and belonging [46,49]. For men, activities that facilitate communication with their spouse or celebrate anniversaries may be beneficial, whereas for women, opportunities to interact with children or grandchildren or share family stories may be particularly impactful.

The fifth topic, “natural comforts,” characterized by keywords such as music, flowers, animals, and natural elements, showed a profound preference for peace and relaxation, emphasizing the importance of surrounding oneself with nature’s tranquility and comfortable environments. This theme held varying degrees of importance among gender-specific clusters, ranking fifth in the men’s cohort and second in the women’s cohort within the themes identified. These findings underscore the need for gender-sensitive care planning of adult daily care preferences, particularly in their care environments. For women, this theme indicated a profound value placed on tranquility and a connection to nature. Integrating features such as garden-related activities and providing rooms with natural views could improve their quality of life and psychological health [50,51]. In contrast, men might prefer different expressions of natural comforts, such as pet therapy or the sounds of a river, which offer a calming and mood-enhancing effect for those who enjoy fishing [52]. Moreover, care strategies could include access to outdoor spaces or nature-themed outdoor activities that align with these identified daily care preferences.

The sixth theme, “emotional and spiritual grounding,” which recognizes humans as spiritual beings, is a critical aspect, despite its relatively low representation in the data. Previous studies have emphasized the importance of emotional and spiritual support for adults, as evidenced by well-being, religion, and comfort [53-55]. A recent mixed methods study on older nursing home residents highlighted the significance of religion and emotional support in their lives [56], further emphasizing the enduring importance of these themes across different settings. Therefore, it is important to understand and integrate an individual’s religious and cultural beliefs and values into the person-centered care plan and care delivery. Introducing care environment elements and tailored activities that align with this theme, such as organizing music sessions with spiritually uplifting songs, establishing areas for reflection or prayer, offering life review programs, or arranging pastoral visits, can contribute to improving adults’ life satisfaction, spiritual well-being, connectedness, and discovery of meaning in life [57,58].

### Limitations

While our exploratory study offered meaningful insights, several limitations should be considered. First, efforts to stratify the sample by gender were hindered by the shortness and small size of the men’s textual data, resulting in pronounced sparsity and challenges in conducting reliable gender-based topic modeling. Consequently, we relied on word frequency analysis to detect patterns and the most discussed topics or concepts within each gender. Furthermore, despite recognizing the importance of including the perspective of transgender individuals in understanding daily care preferences, the limited number of responses from this group did not meet the substantial data requirements necessary for comprehensive topic modeling and examination of gender differences. Future investigations should encompass a larger and more diverse dataset to facilitate a more detailed and comprehensive analysis across the gender spectrum. While our paper mostly represents older adults, expanding the scope to a larger dataset offers opportunities to explore the daily care preferences of young and middle-aged adults, potentially yielding valuable insights through comparative studies. It is crucial to acknowledge that our findings are shaped by the available data and sample size and thus may not be generalizable to different age groups. Recent research endeavors are pivotal to refining our understanding and laying the groundwork for developing personalized care plans that account for individual daily care preferences. In addition, this research identified 6 themes related to the daily care preferences of adults, explored gender differences through the proportion of revealed themes from topic modeling and frequently occurring words in the text data, and recommended building person-centered care plans considering gender differences. However, for more refined person-centered care, future research could collect additional data features, such as health status, functional status, communication preferences, and personal history, to build and use machine learning models to create customized care plans.

### Conclusions

This study explored the daily care preferences of community-dwelling adults and uncovered specific environmental factors that play a crucial role. The identified themes of daily care preferences empower clinicians to enhance health-related quality of life for individuals, allowing for incorporating these insights into personalized care planning. Moreover, recognizing gender differences is also essential when developing person-centered care plans. With these findings, the feasibility of care planning based on personal daily care preferences may be enhanced, further emphasizing the ongoing need to tailor care for adults in the community.

## Appendix

Multimedia Appendix 1 The graphical model for latent Dirichlet allocation. D-plate = For all D document, k-plate = For all K topics, N-plate = For all N words, α = Hyperparameter of the Dirichlet distribution, θ_d = Per-document topic proportion, Z_(d,n) = Per-word topic assignment in document d, w_d = Observed word for document d, β_k = Distributions of the words for a given topic K, η= Hyperparameter of the Dirichlet distribution prior on the β_k.

## Abbreviations

K: number of topics
KNIME: Konstanz Information Miner
LDA: latent Dirichlet allocation
NLP: natural language processing

## Data Availability

The datasets analyzed during this study are not publicly available due to confidentiality and proprietary restrictions, but are available from the corresponding author on reasonable request and with permission from MyDirectives, Inc.
