# Supplementary material for: Understanding Gender-Specific Daily Care Preferences: Topic Modeling Study
Source: J Med Internet Res. 2025 May 29;27:e64160. doi: 10.2196/64160 (PMC12163354; doi:10.2196/64160)
Supplement: Multimedia Appendix 1 [file jmir_v27i1e64160_app1.docx]

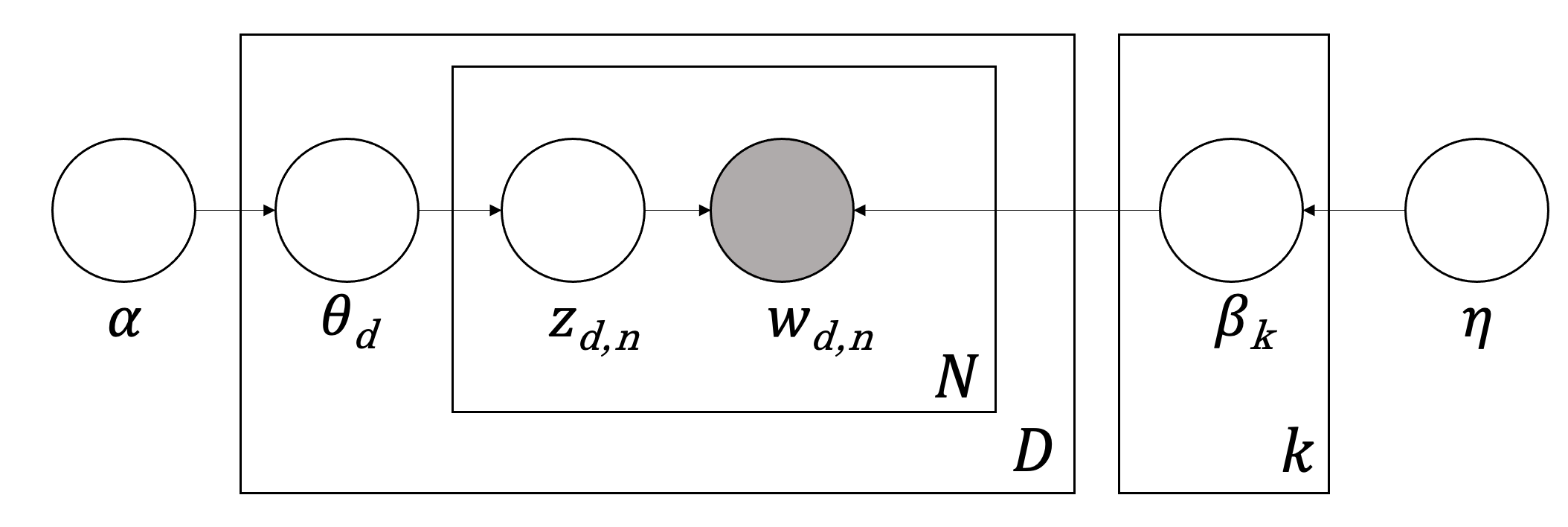


Supplementary 1. The graphical model for latent Dirichlet allocation. *D*-plate = For all *D* document, *k*-plate = For all *K* topics, *N*-plate = For all *N* words, α = Hyperparameter of the Dirichlet distribution, $\theta_{d}$ = Per-document topic proportion, $Z_{d,n}$ = Per-word topic assignment in document *d*, $w_{d}$ = Observed word for document *d*, $\beta_{k}$ = Distributions of the words for a given topic *K*, $\eta$= Hyperparameter of the Dirichlet distribution prior on the $\beta_{k}$.
